# Description of a Double Monster

**Published:** 1874-09-15

**Authors:** J. G. Stokes


### MEDICAL EXAMINER.

A

*Semi-Monthly Journal of Medical Sciences.*

EDITED BY N. S. DAVIS, M.D., AND F. H. DAVIS, M.D.

No. XVIII. CHICAGO, SEPT. 15, 1874. VOL. XV.

### Original Communications.

### DESCRIPTION OF A DOUBLE MONSTER.

By J. G. STOKES, M.D.

MONDAY, July 27th, 1874, I was called to attend a lady, who, in about one hour after my arrival, and without extraordinary or uncommon labor, was safely delivered of a monstrosity, having eight perfectly formed limbs, (four lower and four upper,) one face, one nose, one mouth, and four ears. Although still-born, I am assured by the mother that it was certainly alive up to the evening before its birth.

A careful *post mortem* examination of the brain and viscera of the thorax and abdomen, indicates the following facts:

The union appears to have taken place breast to breast, and face to face, as if the bodies of two children, from the upper portion of the sternum down to near the umbilicus, had been laid open and separated, and then brought together and united, forming after union nearly a perfect square. The thoracic and abdominal cavities are common to both; the thoracic cavity formed by the two, is a hollow square, containing four pleural sacs.

There are two occipital and four parietal bones, but only two frontal; all the other bones of the head, proper, are normal.

The face is well formed; the eyes, nose and mouth are natural in appearance, even handsome; and the ears, which go to form the face, belong, one to each of the beings, while the other two are located directly together, on the opposite side of the head to that upon which the face is located. The bones of the face are normal, as to number and appearance.

The face looks to the right side of one body, and, of course, to the left side of the other. Two vertices are well represented in the head. Looking directly at the spinal column of one, (there being two spinal columns,) can plainly be seen the posterior portion of one head and two ears, which give this *lusus naturæ* a natural appearance; then, turning it around until the other spinal column is presented, the posterior portions of the other head and ears are as plainly to be seen.

One complete hairy scalp covers the entire head.

The neck is larger in proportion than any other part of the body, caused, partly, no doubt, by the upper portion of two spines passing through it.

As has been remarked, there are two spinal columns; but connected with one of them, I find *spina bifida.* This malformation of the spine is far more extensive than in any case that has ever come to my knowledge; the loss of bony structure, beginning at or near the lower dorsal vertebra, and extending down, with total absence of all the lumbar, sacral and coccygeal bones. The cyst covering the malformation was equal, in size, to the child's head. The pubic bone is also absent.

The cerebrum is compounded of two which are inseparable by the most careful dissection. There are, however, separate and distinct cerebella, medullæ oblongatæ, and medullæ spinales.

The four upper extremities are fully and perfectly developed. All the bones of the fingers, hands, arms and fore-arms, and scapulæ are entirely natural. The arms are of equal length and size. All the joints of these four upper extremities are normal. The four shoulders are also perfect, and form a complete square. The hands and fingers are most beautifully formed, and perfect in every respect.

The lower extremities are also natural in all their parts, and of equal length and size.

All the ribs of two well-formed children are present, and there are two perfectly developed clavicles.

The division of the sternums seems to have been complete, extending down through the centre of the ensiform cartilages; the half of one sternum and ensiform cartilage of one child, and the same from the other child, forming one common sternum and ensiform cartilage. Two perfect sternums, formed in this way, are present.

The thorax contains four pleural sacs, enclosing the lungs and hearts of two children, or two pair of lungs and two hearts, all perfectly developed, and natural in appearance. Two mediastina are present, and separate the right from the left lung of each of the beings. As might be expected, there are two tracheæ, but only one œsophageal tube is to be found, and that normal.

The diaphragm is complete, the two being blended into one.

Here one complete stomach is situated just under the diaphragm, and centrally located between the two bodies. Two perfectly formed livers, with two gall bladders and two bile ducts, are here to be seen; both ducts leading into one duodenum. One spleen is discovered, and is located upon the stomach, presenting a natural appearance.

The entire alimentary canal is single. The cavity of the mouth, the œsophagus, the stomach, the duodenum, the jejunum, the ileum, the cecum, the ascending, transverse and descending colon, the sigmoid flexure, the rectum and the aphedra are all in a natural condition. There are three kidneys present, and one urinary bladder, and, necessarily, three ureters leading into the one common bladder. The imperfect child, with "spina bifida" had but one kidney developed, and with this child, or half of the monstrosity, the genitalia and aphedra are entirely wanting, there being not the least trace or appearance of any attempt at development of these organs; while in the other half, the genitalia and aphedra are well developed, and in the natural position. The stomach and bowels are common to both, down to the umbilicus. Below that, the one with "spina bifida" has no abdominal viscera.

It only remains to add that there is extrophy of the urinary bladder, and four separate mammary glands.

There were two funises, both very short. One was severed during parturition, being much the shortest of the two. Both were below the average in size; in fact, the broken funis was much less than any cord that ever came under my observation, not being larger than a crow-quill.

The mother believes that she had not reached the full term by about two weeks; and my opinion is that she is correct. An injury received by her in getting out of a buggy, being, in all probability, the cause of premature labor.

The father and mother are respectable and well-to-do citizens of our town, born and brought up here. The father is a fleshy, robust and fine looking gentleman, about thirty-four years old. The mother is a small and delicately made woman, not weighing, I should suppose, over one hundred pounds. She is well and gracefully formed, and is quite handsome. Her age is about twenty-one. Both of them are entirely free from any hereditary or constitutional taint or disease. Neither on the father's or mother's side of the house have there been any malformations, monstrosities or deformities, as far back as they can trace their history.

---

CHLOROFORM IN STRYCHNINE POISONING (*The New York Medical Record*, July 1, 1874).—A man took five grains of strychnine with a suicidal intent. He was given twenty grains of sulphate of zinc, which produced vomiting. Convulsions had occurred repeatedly, however, and he was seized with one of tetanic form at the time of coming under observation. Every muscle was rigid, and tetanus was complete. Opisthotonos, irregularity of the pulse, varying from 120 to 140 in the minute, with all the accompanying symptoms, were noticeable.

He was immediately placed under the influence of chloroform. The convulsions ceased from the commencement of the anæsthesia, under which the patient was fully kept for three hours. The chloroform was then removed, but the patient did not awake until six hours afterwards,—a case of recovery.

